# Respiratory Microbiota and Health Risks in Children with Cerebral Palsy: A Narrative Review

**DOI:** 10.3390/children12030358

**Published:** 2025-03-14

**Authors:** Pavlina Peneva, Rouzha Pancheva, Silviya P. Nikolova

**Affiliations:** 1 First Department of Internal Disease, Faculty of Medicine, Medical University, 9002 Varna, Bulgaria; 2 Department of Hygiene and Epidemiology, Faculty of Public Health, Medical University, 9002 Varna, Bulgaria; ruja.pancheva@mu-varna.bg; 3 Department of Social Medicine and Healthcare Organization, Faculty of Public Health, Medical University, 9002 Varna, Bulgaria; silviya.p.nikolova@mu-varna.bg

**Keywords:** cerebral palsy, lung, microbiota, respiratory problems

## Abstract

Children diagnosed with cerebral palsy (CP) frequently face a range of intricate health challenges that go beyond their main condition. Respiratory problems represent one of the most crucial factors contributing to morbidity and mortality. This review employed a systematic approach to identify and collate recent findings on the respiratory microbiota in children with CP. The review emphasizes notable microbial alterations in the respiratory systems of children with CP, marked by a decrease in beneficial bacteria (such as *Corynebacterium* spp. and *Dolosigranulum* spp.) and an increase in opportunistic pathogens like *Staphylococcus aureus*, *Pseudomonas aeruginosa*, and *Klebsiella pneumonia.* These changes probably increase the vulnerability of children with CP to frequent respiratory infections, ongoing inflammation, and infections that are resistant to antibiotics. Key factors influencing the composition of microbiota include living in urban areas, socioeconomic factors, seasonal variations, vaccination status, dietary habits, breastfeeding, etc. Although new research has shed significant light on this topic, there are still considerable gaps in our understanding of how these microbial communities develop and interact with the immune responses of the host. Future research should focus on longitudinal studies to track microbiota changes over time and identify interventions that optimize respiratory health in CP.

## 1. Introduction

Children with disabilities, particularly those with cerebral palsy (CP), often experience complex health challenges that extend beyond their primary condition. Among these, respiratory problems are some of the most common and significant, and despite advancements in medical care, they are still the main cause of high morbidity and mortality among the population of children with cerebral palsy [1,2]. Emerging evidence points to the microbiome—the intricate ecosystem of microorganisms, including bacteria, fungi, and viruses—as a key player in shaping respiratory health and disease outcomes.

The human microbiome, composed of approximately 100 trillion microbial cells, colonizes every surface of the body exposed to the external environment, including the skin, gastrointestinal tract (GIT), and respiratory tract [3].

Over millennia, humans and these microbes have coevolved, establishing a symbiotic relationship that significantly influences immune system development and function [4,5].

While the gut microbiome has been extensively studied for its role in various diseases, the respiratory microbiome has only recently become a focus of investigation, facilitated by next-generation sequencing (NGS) technologies. These tools have not only dispelled the longstanding belief in the sterility of the lungs but have also highlighted the importance of “good microbes”, or commensals, in maintaining pulmonary health [6]. The term “microbiota” pertains solely to the specific microorganisms present in a certain area or “niche” of the human body, or “who’s present”. Consequently, since a significant portion of what we label as “microbiome” research typically centers on pinpointing the microbes in a specific environment, “microbiota” may be a more precise term for numerous studies. For the sake of simplicity and in line with standard practice, we will refer to the term “microbiome” to encompass most studies in this review. Moreover, although a microbiome encompasses all types of microbes—viruses, bacteria, fungi, and more—most research to date has concentrated exclusively on bacteria. Consequently, a significant portion of the upcoming discussion concentrates on bacteria, recognizing that further efforts are necessary to define the roles of fungi and viruses in the human microbiome, particularly in the CP children’s microbiome [5].

In healthy individuals, the respiratory microbiota varies between the upper and lower tracts, with bacterial density decreasing in the lower regions. This gradient is believed to result from microaspiration and transient entry of microorganisms from the upper respiratory tract [4,6]. However, in children with disabilities, particularly CP, the respiratory microbiome’s composition and its role in health and disease are poorly understood. CP, the most common physical disability in childhood, affects 1 to 4 per 1000 live births and is associated with various complications, including recurrent aspiration, weakened airway clearance, and impaired lung function [7].

Despite the clear impact of respiratory issues on children with CP, research in this area remains limited [8]. There are so many studies focused on the gut microbiome, gut–lung axis, or gut-brain axis and its complexity in previously healthy children with common lung infections. Existing studies on the respiratory microbiome primarily focus on healthy children or those with conditions such as asthma, cystic fibrosis, ciliary dyskinesia, or pneumonia, leaving a critical gap in knowledge regarding children with disabilities [5,9,10]. This lack of data may be attributed to the complexity of conducting studies in this population, as well as the challenges associated with obtaining representative samples from children with multiple health needs. As a result, much of the existing information on respiratory health in CP children relies on small, heterogeneous cohorts and historical data [11,12,13].

Children with CP face unique challenges that influence their respiratory health, including pseudobulbar syndrome, difficulty swallowing, chronic microaspiration, and the chronic presence of antibiotic-resistant hospital flora. These factors often necessitate empirical approaches to antibiotic treatment, which can lead to inefficacy and prolonged recovery times [14]. Understanding the respiratory microbiota’s role in this context could pave the way for targeted interventions that optimize treatment and improve outcomes for this vulnerable group.

This narrative review explores the current research on the respiratory microbiota in children, emphasizing its composition, the factors that influence it, and its potential implications for children with CP. The review delves into how various environmental, lifestyle, and genetic factors shape the respiratory microbiota and highlights the unique challenges that children with CP may face in this context. By addressing existing gaps in the literature, this work seeks to provide a deeper understanding of the interactions between the microbiome and the specific health issues encountered by children with disabilities. Ultimately, the review aims to inform more effective diagnostic and therapeutic strategies for managing respiratory health in this vulnerable population.

## 2. Methods

This review adopted a systematic approach to identify and synthesize recent research on the respiratory microbiota in children, specifically focusing on those with cerebral palsy (CP). A comprehensive search was conducted across two major scientific databases, PubMed and Scopus, between September 2024 and October 2024. The search utilized combinations of the keywords “lung microbiome OR lung microbiota” and “nasopharyngeal microbiome OR nasopharyngeal microbiota”, alongside the term “children”. To ensure the relevance and timeliness of the results, only studies published in English from 2019 to October 2024 were considered for inclusion.

The inclusion criteria were explicitly defined to focus on studies examining the lung or nasopharyngeal microbiota in children with CP. Eligible studies were required to be original research exploring microbiota composition, investigating risk factors influencing respiratory microbiota in this population, or involving children with CP as part of conditions affecting respiratory health. Studies were considered if they involved participants under 18 years of age and were fully accessible in English. Articles that did not meet these criteria—such as those addressing unrelated populations, lacking full access, or published outside the designated time frame—were excluded.

**Figure 1:**
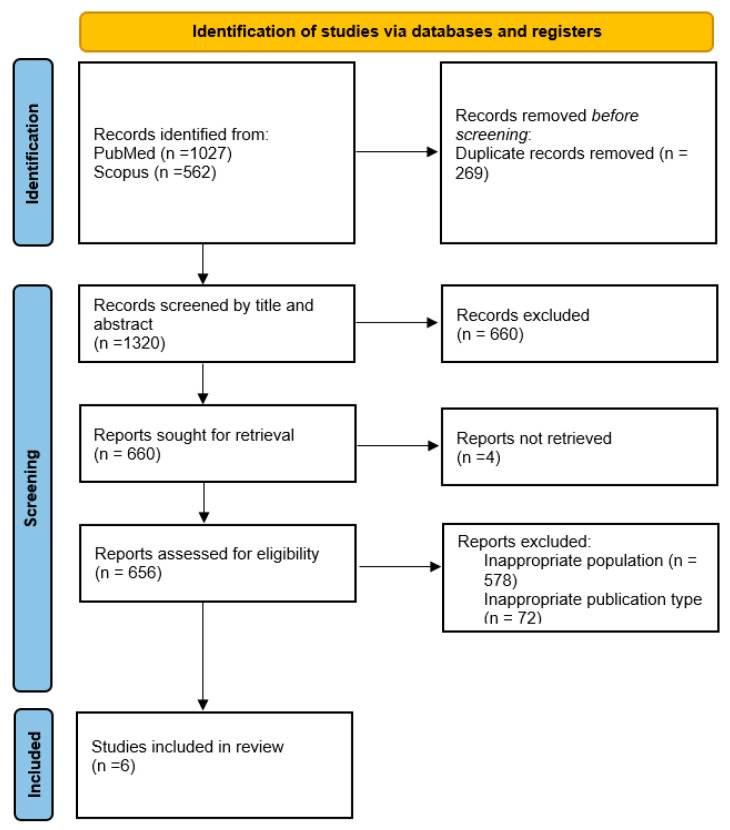
Flowchart of the study selection process.

The initial search yielded 1589 articles. After screening titles and abstracts, 660 studies were excluded due to their lack of relevance to the review’s focus. Four full-text articles were excluded due to language limitations in the full text, leaving 656 articles for in-depth evaluation. Following a comprehensive review of these, 650 studies were excluded for not meeting the inclusion criteria. In total, six studies were selected for inclusion in this review (Figure 1).

Key details from the selected studies—including study design, participant demographics, microbiota composition, influencing factors, and findings specific to CP—were extracted and summarized and are presented in Table S1.

## 3. Results

For this review article, six studies spanning the years 2019 to 2024 were included. Four of these studies come from industrialized high-income countries, such as Belgium, the United Kingdom, and China. Two of the included studies were conducted in upper-middle-income countries, such as Turkey and Ukraine.

Two investigations study the microbiological compound of the upper or lower respiratory tract using a deep smear from the oropharynx [15] or throat swabs [16]. 16S ribosomal ribonucleic acid (rRNA) sequencing was used to study core supragingival plaque microbiota [17], gut microbiota, and oral microbiota [18]. Retrospectively, only one of the studies used videofluoroscopic swallowing studies [14], and the other one used microbiology lab records [19]. One of the follow-ups studied the respiratory microbiota in order to optimize antibacterial therapy of recurrent respiratory diseases [15].

### 3.1. The Upper Respiratory Tract Microbiota

Microbiological research of the upper respiratory tract, using a deep oropharyngeal smear, revealed a predominance of *Pseudomonas aeruginosa* (37.5%) and intestinal opportunistic microflora (43.7%), such as *Klebsiella pneumoniae* and *Proteus vulgaris*. Frequent combinations were observed with *Streptococcus pyogenes* (50%), *Staphylococcus aureus* (50%), and *Candida* spp. (37.5%). Opportunistic flora was identified in 43.7% of children, often accompanied by a reduction or near absence of normal microflora, including *Streptococcus* spp., *Neisseria* spp., and *Aerococcus viridans* [15].

Using 16S rRNA gene sequencing, analysis of the oral microbiota in children with cerebral palsy and epilepsy (CPE) revealed that *Prevotella* (15.4%), *Fusobacterium* (9.3%), and *Neisseria* (7.6%) were the three most abundant genera. These findings suggest potential links to caries, periodontitis, and malnutrition. In comparison to healthy children, *Firmicutes* and *Bacteroides* were significantly lower in CP children, while *Actinomycetes* showed a marked increase. Additionally, the oral and gastrointestinal microbiota were closely correlated, with notable differences in bacterial composition in CP children, including *Bifidobacterium, Fusobacterium, Bacteroides*, and *Neisseria* [18].

Supragingival plaque samples were collected for 16S rRNA sequencing and revealed that “the core microbiota” of the supragingival plaque in CP children with caries were *Prevotella, Fusobacterium*, *Campylobacter*, *Leptotrichia*, *Porphyromonas*, *Saccharibacteria*, *Actinomyces*, *Catonella*, *Alloprevotella*, *Capnocytophaga*, *Parvimonas*, *Streptobacillus*, *Peptostreptococcaceae*, *SR1*, and *Lachnoanaerobaculum*. Their total relative abundance accounted for 74.4% in severe caries, 81.4% in moderate caries, and 77.8% in caries-free CP children. Prevotella, Alloprevotella, and Streptobacillus were enriched with greater severity of caries, whereas the relative abundances of Campylobacter and Capnocytophaga were decreased [17].

### 3.2. The Lower Respiratory Tract Microbiota

Using the throat swabs to evaluate lower airway microorganisms, 28 patients (35%) were found to have at least one positive respiratory culture. Only 4 patients (5%) were infected with *Ps. aeruginosa*. Gram-negative bacteria were isolated in 22% of the positive throat swabs, *S. aureus* was found in 14%. There was a correlation between the Gross Motor Function Classification System (GMFCS) score and colonization with gram-negative bacteria. The prevalence of *Ps. aeruginosa* in children with CP is low, and gram-negative bacteria were most commonly found in patients with higher GMFCS scores III, IV, and V [16].

Retrospectively, identifying the microbiology lab records revealed no significant association between the diagnosis of CP or neuromuscular diseases (NMD) and *Ps. aeruginosa* respiratory infections. Of the 25 patients with *Ps. aeruginosa* isolates, 19 (76%) had NMD, and 6 (24%) had CP. However, there was a significant association between the presence of a tracheostomy and *Ps. aeruginosa* positive samples (*p* < 0.05). The majority of the patients (15%) with respiratory *Ps. aeruginosa* isolates did not significantly deteriorate clinically, and outcomes in relation to antibiotic treatment choices were unclear due to small patient numbers [19].

Videofluoroscopic swallowing studies found that CP children with severe gastroesophageal reflux during the oral feeding stage (before switching to a nasogastric tube (NGT)), had a higher hospitalization rate and duration due to respiratory infections than the children with NGT who had been hospitalized twice or less yearly (0.12 vs. 0.17/0.005106 vs. 0.005353). At the same time, *Ps. aeruginosa* is a leading pathogen (47.1%), followed by *Acinetobacter baumannii* (26.5%), and *Klebsiella* spp. (17.6%) [14].

The most commonly observed bacteria in the various parts of the respiratory system in children with cerebral palsy are summarized and reflected in Table S2.

## 4. Discussion

**Figure 2:**
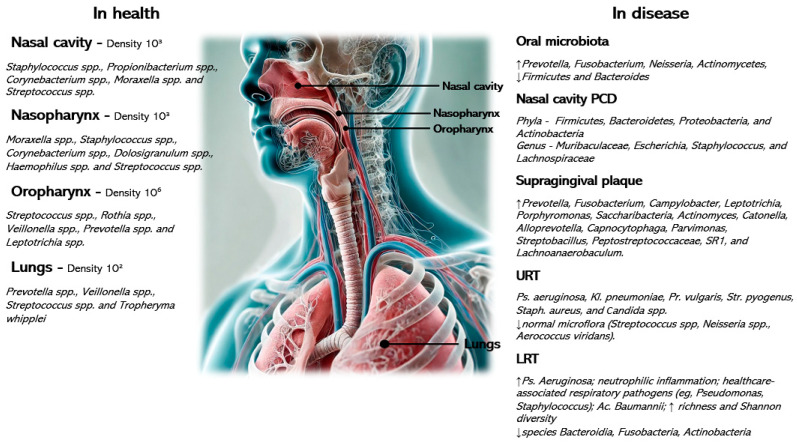
Microbial gradients along the respiratory tract in health and disease with the associated most relevant causative agents.

This review highlights distinct microbial shifts in the respiratory tracts of children with cerebral palsy (CP) [14,15,16,17,18,19] (Figure 2), characterized by a depletion of commensal bacteria (e.g., *Corynebacterium* spp., *Dolosigranulum* spp.) and an overrepresentation of opportunistic pathogens such as *Staphylococcus aureus*, *Pseudomonas aeruginosa*, and *Klebsiella pneumoniae* [16,20].

Physiological and microbial gradients exist along the nasal cavity, nasopharynx, oropharynx, trachea, and lungs. Factors such as pH, humidity, temperature, partial pressures of oxygen or carbon dioxide, and size of particles might influence the density of microorganisms, which gradually decreases along the respiratory tract. In the event of inflammation of the respiratory tract or in the presence of additional factors, such as oropharyngeal aspiration, poor oral hygiene, nutrition, or other factors typical of children with cerebral palsy, disruption and/or replacement of the normal flora by pathological flora is observed [7,9,15].

These changes likely contribute to the increased susceptibility of CP children to recurrent respiratory infections, chronic inflammation, and antibiotic-resistant infections [2,14,19]. Recurrent respiratory diseases in CP often result in prolonged hospitalizations, diminished lung function, and heightened morbidity. The findings emphasize the necessity for targeted interventions, including microbiota-informed antibiotic regimens, prophylactic strategies, and alternative therapies such as probiotics [21].

Recent applications of next-generation sequencing have revealed that the respiratory tract harbors diverse bacterial communities, refuting the traditional assumption of lower airway sterility [22]. Multiomics techniques, including metagenomics and metabolomics, provide insights into microbial composition and functional interactions, offering potential biomarkers for respiratory health monitoring in CP children [23,24].

Microbial colonization in the upper respiratory tract (URT) begins at birth, influenced by maternal transmission and environmental factors. *Corynebacterium* spp. and *Dolosigranulum* spp. dominate in vaginally delivered infants, whereas cesarean section births favor *Staphylococcus aureus* and anaerobes such as *Prevotella and Veillonella* [23]. By 6 months, *Moraxella* spp. emerges as a key commensal, promoting respiratory homeostasis [7].

A study using a microbiota-based machine-learning algorithm found that infants had higher RTIs in the first year of life due to an altered microbial developmental trajectory, resulting in decreased community stability, reduced microbial community, early *Moraxella* enrichment, and later *Neisseria and Prevotella* spp. [23]. Disruptions in this trajectory, often due to antibiotic exposure, malnutrition, crowding, frequent infections, or access to healthcare, contribute to long-term respiratory complications in CP children [7,25].

Other key factors are socioeconomic status, urban living conditions, seasonal variations, vaccination status, dietary habits, presence of siblings, attendance at day-care, exposure to smoke, and previous infections, highlighting the dynamic nature of the microbiota in early life shaped by various host and environmental influences [25,26,27,28]. Socioeconomic factors play a crucial role in shaping the respiratory microbiota and health outcomes of children with CP. Those from disadvantaged backgrounds face a higher risk of severe respiratory morbidity, largely due to limited access to healthcare, higher rates of malnutrition, and greater exposure to environmental pollutants [29,30]. Additionally, lower maternal education and poorer living conditions contribute to more severe comorbidities, including respiratory issues, in children with CP [31]. These socioeconomic disparities are also linked to alterations in respiratory microbiota, increasing susceptibility to frequent and severe respiratory infections [23].

Breastfeeding supports immune resilience by transferring beneficial microbes like *Bifidobacterium* spp. and *Lactobacillus* spp., whereas early-life antibiotic use can diminish microbial diversity, increasing infection susceptibility [25,32].

On the other hand, worldwide, there has been an increase in multidrug resistance (MDR), which is regarded as a hazard to public health. Several recent investigations reported the emergence of MDR-bacterial pathogens from humans. *E. coli* is an opportunistic pathogen in the gut of healthy individuals. Some strains have the potential to overcome host defense systems and colonize other tissues and host organs. In this way, they become extraintestinal pathogenic *E. coli*. As a result, individuals who were previously healthy or who are vulnerable—such as children with cerebral palsy—may develop a number of diseases, including pneumonia. Numerous virulence genes encode different virulence factors, such as adhesins, toxins, iron-acquisition factors, and invasins, which are present in strains of *E. coli*. Unfortunately, antimicrobial resistance is spreading alarmingly. The majority of strains develop into MDR *E. coli* as a result of the research’s geographic scope, variations in antibiotic prescribing patterns, and certain nations’ absence of an extensive monitoring mechanism for the proper use of antibiotics [33]. These insights underscore the importance of optimizing environmental and dietary factors to maintain respiratory microbiota stability in CP children. The bidirectional gut–lung axis suggests that gut microbiota alterations can influence respiratory health, with systemic inflammation and immune dysregulation playing mediating roles [34]. While well-documented in conditions like asthma and cystic fibrosis, the gut–lung connection in CP remains underexplored. Future studies should investigate how enteric dysbiosis in CP children affects pulmonary outcomes [3,10].

Functional constipation is a common problem in children, particularly in children with CP. Though constipation can have various causes, changes in gut flora, including the rise or fall of certain colonic microbiota species, are thought to potentially contribute to the development of functional constipation in children. To assess which probiotic strain could be beneficial in treatment approaches for functional constipation, a thorough understanding of the gut microbiota composition is essential. Up to now, probiotics, especially *Lactobacillus* species, have been utilized in numerous randomized controlled trials to address different gastrointestinal issues, such as functional constipation. The possible link between functional gastrointestinal disorders and probiotics is compelling, presenting intriguing therapeutic and preventive options in probiotic and gastrointestinal research. Enhanced gastrointestinal motility was seen in certain tests with specific strains of *Lactobacillus*, particularly *L. reuteri* and *L. acidophilus*, as well as *B. bifidum*. However, other studies found no significant differences between the prevalence of intestinal *Lactobacillus* species in healthy and constipated children. Moreover, they found that in the healthy subjects and constipated group, *L. paracasei* and *L. planetarium* species were predominant types of lactobacilli [35]. Several systematic reviews and meta-analyses do not recommend the routine use of probiotics in the treatment of constipation [36,37], but there are no clear guidelines in CP children.

Respiratory pathogens gain entry via inhalation, aspiration, or translocation from the gastrointestinal tract. The presence of opportunistic flora at high bacterial loads (≥10^6^ CFU/m^3^) is strongly linked to bronchopulmonary disease [38]. However, few studies have characterized the microbial signatures of CP children with severe neurological impairments, limiting precision in treatment approaches [15,39]. Sleep disturbances are prevalent in CP children due to upper airway obstruction, poor pharyngeal muscle tone, and oropharyngeal aspiration [40]. Adenotonsillar hypertrophy further disrupts microbial homeostasis, reducing beneficial commensals like *Corynebacterium* and *Moraxella* while favoring the overgrowth of *Granulicatella*, *Streptococcus*, and *Haemophilus* species. It was found that the pharyngeal and nasal microbiota of children with adenotonsillar hypertrophy were similar. There was a significant decrease in microbial richness in the pharynx of adenotonsillar hypertrophy children in comparison with healthy ones. By β-diversity analysis, the differences in the pharyngeal ecosystem between both groups were confirmed. Linear discriminant analysis effect size showed an increase of the genera *Granulicatella*, *Streptococcus*, *Staphylococcus*, *Neisseria*, and *Haemophilus*, as well as a reduction of *Corynebacterium*, *Dolosigranulum*, and *Moraxella* in pharyngeal swabs of adenotonsillar hypertrophy patients. In the same way, the nasal microbiota of the adenotonsillar hypertrophy group exhibited a unique microbial signature in comparison to healthy children. Nevertheless, in contrast to the pharyngeal microbiota, this was marked by a rise in microbial richness. Despite some variations, the nasal microbiome markers resembled those found in the pharyngeal microbiota. Specifically, it noticed a rise in the *Rothia*, *Granulicatella*, *Streptococcus*, *Neisseria*, and *Haemophilus genera* and a decrease in *Corynebacterium*, *Pseudomonas*, *Acinetobacter*, and *Moraxella* in patients with adenotonsillar hypertrophy. Additionally, administering probiotic spray containing a minimum of 125 × 10^9^ colony-forming units (CFU)/g of lyophilized *Streptococcus salivarius 24SMBc* and *Streptococcus oralis* 89a led to a more significant decrease in the growth of various bacteria in both the pharynx (*S. salivarius*, *S. aureus*, *G. haemolysans*, *N. subflava*, *S. parasanguis*, *H. influenzae*, and *E. cloacae*) and the nose *(H. influenzae*, *S. aureus*, *S. salivarius*, *S. oralis*, *S. vestibularis*, *E. cloacae*, and *N. subflava*). These findings indicate that probiotics were able to inhibit the growth of certain harmful bacteria, specifically H. influenza [21], and surgical and microbiome-targeted interventions may mitigate airway complications [41].

Future clinical trials should assess the long-term benefits of probiotic formulations in reducing respiratory disease burden in CP populations. *Streptococcus pneumoniae* significantly alters nasopharyngeal microbiota diversity, increasing colonization by opportunistic bacteria like *Staphylococcus* and *Moraxella* [41]. Understanding these interactions may inform vaccine strategies and antibiotic stewardship programs tailored for CP children to prevent chronic infections [26].

Children with CP exhibit a higher prevalence and severity of dental caries. While researchers have investigated various risk factors associated with caries in children with CP, the role of microorganisms in the development of caries remains a crucial area for further research. Notably, a distinct composition of supragingival plaque microbiota has been identified between children with CP who have severe caries and non-CP children with severe caries. *Prevotella*, *Fusobacterium*, *Campylobacter*, *Leptotrichia*, *Porphyromonas*, *Saccharibacteria*, *Actinomyces*, *Catonella*, *Alloprevotella*, *Capnocytophaga*, *Parvimonas*, *Streptobacillus*, *Peptostreptococcaceae*, *SR1*, and *Lachnoanaerobaculum* were “the core microbiota” of the supragingival plaque in CP children with caries, while the maintenance of *Capnocytophaga* and *Campylobacter* in oral microbiota might be essential to promote the caries-free condition in CP children [17]. Likewise, *Actinomycetes*, *Prevotella*, *Fusobacterium*, and *Neisseria* were increased significantly in CP and epilepsy children and were the top four abundant genera, representing the oral microbiota and suggesting potential correlations with caries, periodontitis, and malnutrition [17,18]. Given the established gut–lung connection, dysbiosis in oral flora may exacerbate pulmonary conditions [34]. That is why integrating dental hygiene interventions into CP respiratory management strategies warrants further exploration [17,18].

Bronchopulmonary dysplasia (BPD), prevalent in preterm infants, shares microbial disruptions with CP-associated respiratory conditions. Increased expression of *Prevotella* and decreased *Caulobacter* abundance in BPD have been linked to disease severity [42]. Understanding these microbial patterns in CP children with early lung injury may provide insights into long-term pulmonary outcomes [42,43].

Severe gastroesophageal reflux and profound motor impairment are key predictors of bronchiectasis in CP [14,40]. While bacterial colonization is well-documented, metagenomic studies are needed to delineate the specific microbial contributions to bronchiectasis progression in CP children [44].

Tracheostomized CP children exhibit distinct microbial communities, with reduced diversity and increased colonization by *Pseudomonas aeruginosa* and *Staphylococcus aureus*. Chronic inflammation, driven by neutrophil activation and oxidative stress, further exacerbates airway damage [39,45]. Understanding the microbiome shifts associated with tracheostomy could inform individualized infection control strategies [46].

Studies utilizing 16S rRNA metagenomics have revealed microbial resilience in the upper and lower respiratory tracts, influencing disease susceptibility [47]. However, obtaining lower airway samples in non-intubated CP children remains ethically and logistically challenging [48]. Innovations in non-invasive sampling and biomarker identification could improve diagnostic capabilities [48,49]. The implementation of molecular methods with syndromic panels has the potential to be a powerful decision-making tool for patient management despite requiring appropriate use of the test in different patient populations. The timing of collection, transportation, and storage of samples are crucial for accurate microbiological diagnosis and interpretability of results. For respiratory tract infections, specific specimens, collection methods, and storage conditions are required. Nasopharyngeal washes, aspirates, swabs, oropharyngeal swabs, and combined swabs are recommended for detecting respiratory viruses. Nasopharyngeal aspirates and nasal washes are difficult to collect in clinical practice due to the need for specific suction devices and skilled operators. Nasopharyngeal or oropharyngeal swabs are easier and painless and can be performed outside the hospital. Lower respiratory tract specimens include spontaneous sputum, bronchoscopy, endotracheal aspirates, and transthoracic lung aspiration. Collection is limited to severe cases and invasive techniques, with bronchoalveolar lavages being the most commonly used. Different methods for confirmation of viral or microbiological diagnosis are used in practice: microscopy, blood or sputum culture, rapid immunoassays, serological tests, and nucleic acid amplification tests. However, advanced molecular diagnostic technologies can revolutionize microbiological diagnoses in clinical labs, making them faster and more robust. Multiplex respiratory testing, despite its high cost, can limit unnecessary testing and minimize patient costs. Early administration of targeted antibiotic therapy and rapid adjustment of empirical therapy can improve CP children’s outcomes [50].

## 5. Limitations and Strengths

This review is limited by the heterogeneity of study methodologies, sample sizes, and geographic variations in respiratory microbiota composition, which complicates drawing definitive conclusions. Next, our study covers publications on the respiratory microbiota in children with cerebral palsy in the last 5 years, when the information is more complete and precise, based on modern tests such as next-generation sequencing technologies. Furthermore, due to the lack of sufficient data, the exclusion of viral and fungal microbiota restricts a comprehensive understanding of respiratory dysbiosis in children with cerebral palsy (CP). While existing studies primarily focus on the bacterial microbiota, further investigation is needed to explore the roles of viral and fungal components, which could provide additional insights into respiratory health in this population.

Future research should prioritize longitudinal studies that incorporate multiomics approaches to better elucidate the causal relationships between microbiota disruptions and respiratory outcomes in CP children [51].

Additionally, understanding these relationships could inform microbiome-targeted interventions, precision antibiotic therapies, and the integration of microbiota monitoring into clinical care [52]. Addressing these challenges could pave the way for improved respiratory health management in CP children, ultimately enhancing their quality of life [13]. The limited number of studies underscores the urgent need for multicenter, longitudinal research to better understand respiratory microbiota dynamics in children with CP, incorporating larger and more diverse cohorts. Future studies should adopt standardized methodologies, including consistent use of next-generation sequencing technologies and uniform diagnostic criteria, to enable better comparability across studies.

## 6. Conclusions

This review underscores the critical role of respiratory microbiota in shaping health outcomes for children with CP, as well as the difficulties in collecting and validating microbiological or virological results. The growing body of evidence suggests that microbial composition in the upper and lower airways is influenced by a complex interplay of physiological, environmental, and therapeutic factors. While emerging research has provided key insights, significant gaps remain in understanding how these microbial communities evolve and interact with host immune responses. Addressing these gaps requires a multidisciplinary approach that integrates microbiome-targeted therapies, precision antibiotic use, and preventive strategies such as probiotics and vaccination. Future research should focus on longitudinal studies to track microbiota changes over time and identify interventions that optimize respiratory health in CP. By refining diagnostic approaches and treatment strategies, we can work toward reducing the burden of respiratory complications and improving the overall quality of life for children with CP.

## Data Availability

No new data were created during this study.

## Supplementary Materials

The following supporting information can be downloaded at: https://www.mdpi.com/article/10.3390/children12030358/s1, Table S1: Details from the selected studies—study design, participant demographics, microbiota composition, influencing factors, and findings specific to CP. References [14,15,16,17,18,19] listed in Table S1; Table S2: Presentation of beneficial and non-beneficial microbes in CP children.
