# Supplementary material for: Respiratory Microbiota and Health Risks in Children with Cerebral Palsy: A Narrative Review
Source: Children (Basel). 2025 Mar 14;12(3):358. doi: 10.3390/children12030358 (PMC11941506; doi:10.3390/children12030358)
Supplement: Supplementary file 1 [file children-12-00358-s001.zip › children-3488778-supplementary.pdf]

Table S1. *Details from the selected studies — study design, participant demographics, microbiota composition, influencing factors, and findings specific to CP. References (14-19) listed in Table S1.*

Publication information						Participants information						Results		
						Patients			Controls					
**Authors**	**Title**	**Year**	**Country**	**Source (one/multi center)**	**Aim/Study criteria**	**(N) Total**	**Age Mean/ Range**	**Gender of the participants**	**(N) Total**	**Age Mean/ Range**	**Gender of the participants**	**Bacterial diversity**	**Type of analysis**	**Reported genera/ Conclussion**
**Kürtül ÇM et al.** [14]	The respiratory problems of patients with cerebral palsy requiring hospitalization: Reasons and solutions	2020	Turkey	Department of Pediatrics, Ankara Child Health and Diseases Hematology Oncology Training and Research Hospital, Ankara City	Retrospective analyse of CP patients who were followed-up and treated due to respiratory problems requiring hospitalization were examined, and factors affecting the frequency and duration of hospitalization in wards and in the intensive care unit;	83 CP	NA	NA	NA	NA	NA	Pseudomonas aeruginosa (47.1%), Acinetobacter baumannii (26.5%), Klebsiella spp. (17.6%), Stenotrophomonas maltophilia (8.8%), Staphylococcus aureus (8.8%), Candida spp. (5.9%), Corynebacterium spp. (5.9%), and Serratia spp. (2.9%)	Videofluoroscopic swallowing studies	Patients hospitalized more than two times during the oral feeding period, before switching to nasogastric tube, had higher hospitalization rate, and duration than the patients who had been hospitalized twice or less (0.12 vs. 0.17/0.005106 vs. 0.005353). Pseudomonas aeruginosa is a leading pathogen (47.1%), followed by Acinetobacter baumannii (26.5%), and Klebsiella spp. (17.6%).
**Koreniuk O.** [15]	Respiratory microbiota disorders in children with neurological disabilities at repeated respiratory diseases	2021	Ukraine	The Children's Clinical Hospital No. 6 in Dnipro in 2017–2019	To study the respiratory microbiota to optimize antibacterial therapy of recurrent respiratory diseases; 16 children from the nursing home for the disabled aged 4 to 12 years with	16	4-12 years	NA	NA	NA	NA	Gr-negative flora in 43.7 % (n=7) patients with a predominance of conditionally pathogenic intestinal flora (Klebsiella pneumoniae, Proteus vulgaris, E. coli). Pseudomonas 37.5 % (n=6) of cases. Staphylococcus aureus in 50 % (n=8) cases, Streptococcus pyogenus in 50 % (n=8) cases), Candida in 37.5 % (n=6) cases.	The microbiological research of the upper respiratory tract using a deep smear from the oropharynx	The upper respiratory tract had a dominance of Pseudomonas aeroginosa, intestinal opportunistic microflora (Klebsiella pneumoniae, Proteus vulgaris) and their frequent combination with Streptococcus pyogenus, Staphylococcus aureus and yeast-like mushrooms of the Candida genus. Opportunistic flora 43.7 % of children, against the background of a decrease or practically absence of normal microflora (Streptococcus spp,

					severe neurological pathology;									Neisseria spp., Aerococcus viridans). High resistance of the identified microorganisms to most medicines.
**Romaen K et al.** [16]	Pseudomonas aeruginosa in children with cerebral palsy: a prospective study	2023	Belgium	The Antwerp Reference Centre of Cerebral Palsy in the Antwerp University Hospital	The prevalence of Ps. aeruginosa (PA) and its association with respiratory disease in a prospective study including patients, aged 0–18 years, with a diagnosis of CP who attended either specialized	79	0-18; 8.44	37M:42F	NA	NA	NA	28 patients (35%) had at least one positive respiratory culture. Only 4 patients (5%) were infected with Ps. aeruginosae. Gram negative bacteria were isolated in 22% of the positive throat swabs, S. aureus was found in 14%. Most pathogens were found in patients with higher GMFCS score (GMFCS III, IV and V).	Throat swabs were used to evaluate lower airway microorganisms	The prevalence of Ps. aeruginosa in children with CP was low, gram-negative bacteria were most commonly found. No statistically significant relation was found between the number of colds and/or pneumonia, the use of antibiotics or hospitalization, and having a positive culture, colonization with S. aureus, P. aeruginosa or gram-negative bacteria. The prevalence of respiratory tract infections is against increasing in the post-covid era.
**Liu M. et al.** [17]	From Mouth to Brain: Distinct Supragingival Plaque Microbiota Composition in Cerebral Palsy Children With Caries	2022	China	The Department of Neurology, Maternity and Child Healthcare Hospital Longgang district, Shenzhen	Establishing the Complex Networks of Core Supragingival Plaque Microbiota Correlation Among CP Children With Different Caries Severities	55 CP	10.86 ± 2.28 years	8M:14F	23 non-CP	10.17 ± 1.99 years	8M:15F	Prevotella, Fusobacterium, Campylobacter, Leptotrichia, Porphyromonas, Saccharibacteria, Actinomyces, Catonella, Alloprevotella, Capnocytophaga, Parvimonas, Streptobacillus, Peptostreptococcaceae, SR1, and Lachnoanaerobaculum	Supragingival plaque samples were collected for 16S rRNA sequencing	"The core microbiota" of the supragingival plaque in CP children with caries were Prevotella, Fusobacterium, Campylobacter, Leptotrichia, Porphyromonas, Saccharibacteria, Actinomyces, Catonella, Alloprevotella, Capnocytophaga, Parvimonas, Streptobacillus, Peptostreptococcaceae, SR1, and Lachnoanaerobaculum, with total relative abundance accounting for 74.44% in CP severe caries, 81.40% in CP moderate caries, and 77.85% in CP caries-free children.
**Congfu Huang et al.** [18]	Correlations between gastrointestinal and oral microbiota in children with cerebral	2022	China	Department of Pediatrics, Longgang District Maternity & Child Healthca	Correlation between gut and oral microbiota (OM) in children with cerebral	27 CP	4-14 years	NA	NA	NA	NA	The abundance of Bacteroidetes (27.808%), Proteobacteria (23.655%), Firmicutes (15.681%), Actinobacteria (13.186%), and Fusobacteria (10.905%) was the highest in the OM. In the GM the top 5 were Firmicutes (averaged 32.689%),	Gut microbiota (GM) and oral microbiota (OM) through 16S rRNA gene	Prevotella, Fusobacterium, and Neisseria were the top three abundant genera of the OM and suggesting potential correlations with caries, periodontitis, and malnutrition. Firmicutes and

	palsy and epilepsy			re Hospital, Shenzhen, China, The social welfare center of Longgang District	palsy and Epilepsy							Bacteroidetes (28.869%), Actinobacteria (27.547%), Proteobacteria (4.698%), and Fusobacteria (1.903%)	sequencing	Bacteroides in the oral cavity were significantly lower in CPE children than in healthy children, whereas the abundance of Actinomycetes increased significantly in CPE children. The OM and GM correlated with each other closely, and the bacterial components of these microbiota in CPE children were remarkably different from those in healthy children, such as Bifidobacterium, Fusobacterium, Bacteroides, and Neisseria.
**Gregson E. et al.** [19]	Pseudomonas aeruginosa infection in respiratory samples in children with neurodisability—to treat or not to treat?	2021	UK	Department of Respiratory Medicine, Sheffield Children'sNHS Foundation Trust, Sheffield, UK	To investigate the prevalence of Ps, aeruginosa (PA) in patients with complex neurodisability (children with neuromuscular disease (NMD) and cerebral palsy GMFCS 4 or 5), a 12-month retrospective study	162 (NMD)	11,7 years	99M:63F	113 CP,	11,7 years	NA	Of the 25 patients with Ps. aeruginosa isolate, 19 (76%) had NMD and 6 (24%) had CP; The majority of the 15% patients with respiratory Ps. aeruginosa isolates did not significantly deteriorate clinically and outcomes in relation to antibiotic treatment choices were unclear due to small patient numbers.	158 positive samples for Ps. aeruginosa were identified in the microbiology lab records	There was no significant association between diagnosis of NMD or CP and Ps. aeruginosa analysis. There was no significant association between use of NIV or gastrostomy and Ps. aeruginosa analysis, but there was a significant association between presence of a tracheostomy and Ps. aeruginosa positive samples (p<0.05).

Table S2. *Presentation of beneficial and non-beneficial microbes in CP children*

Conditions	Beneficial microbes	Non-Beneficial microbes
**Breastfeeding**	↑*Bifidobacterium spp. Lactobacillus spp.*	
**Upper respiratory tract mictobiota in children soon after delivery**	*Corynebacterium spp., Dolosigranulum spp.* - in vaginally *delivered infants;* *Staph. aureus and anaerobes /Prevotella, Veillonella/*- in cesarean section delivered	↑ *Moraxella, Neisseria* and *Prevotella spp.*
**Oral microbiota in CP children and epilepsy**	↓Firmicutes ↓Bacteroides	↑*Actinomycetes, Prevotella, Fusobacterium,* and *Neisseria* top four abundant genera, suggesting potential correlations with caries, periodontitis, and malnutrition
**Oropharyngeal micribiota and neurological disabilities /+CP/**	↓normal microflora, including *Streptococcus spp., Neisseria spp., and Aerococcus viridans*	↑*Ps. aeruginosa, and intestinal opportunistic microflora (Kl. pneumonia, Pr. vulgaris), Str. pyogenes,, Staph. aureus, and Candida spp.*
**Pharyngeal microbiota in adenotonsillar hypertrophy**	↓ *Corynebacterium, Dolosigranulum, and Moraxella*	↑*Granulicatella, Streptococcus, Staphylococcus, Neisseria, and Haemophilus*
**Supragingival plaque in CP children with caries**	↑*Capnocytophaga and Campylobacter* promote the caries-free condition in CP children	↑"The core microbiota" - *Prevotella, Fusobacterium, Campylobacter, Leptotrichia, Porphyromonas, Saccharibacteria, Actinomyces, Catonella, Alloprevotella, Capnocytophaga, Parvimonas, Streptobacillus, Peptostreptococcaceae, SR1, Lachnoanaerobaculum*
**LRT infections in BPD/CP**	↑*Caulobacter abundance*	↑*Prevotella*
**Tracheostomized CP children**	↓ diversity	↑*colonization by Ps. aeruginosa and Staph. aureus*
**CP children with severe gastroesophageal reflux**		↑*Ps. aeruginosa, Ac. baumannii, Klebsiella spp.*
**Gastrointestinal motility**	*Lactobacillus /L. reuteri* and *L. acidophilus, B. bifidum/* enhance gastrointestinal motility	
